# NBLAST: a graphical user interface-based two-way BLAST software with a dot plot viewer

**DOI:** 10.5808/gi.22053

**Published:** 2022-09-30

**Authors:** Beom-Soon Choi, Seon Kang Choi, Nam-Soo Kim, Ik-Young Choi

**Affiliations:** 1 BIT Institute NBIT Co., Ltd., Chuncheon 24341, Korea; 2 Department of Agriculture and Life Industry, Kangwon National University, Chuncheon 24341, Korea

**Keywords:** BLAST, database, dot plot, sequence alignment, two-way BLAST system

## Abstract

BLAST, a basic bioinformatics tool for searching local sequence similarity, has been one of the most widely used bioinformatics programs since its introduction in 1990. Users generally use the web-based NCBI-BLAST program for BLAST analysis. However, users with large sequence data are often faced with a problem of upload size limitation while using the web-based BLAST program. This proves inconvenient as scientists often want to run BLAST on their own data, such as transcriptome or whole genome sequences. To overcome this issue, we developed NBLAST, a graphical user interface-based BLAST program that employs a two-way system, allowing the use of input sequences either as “query” or “target” in the BLAST analysis. NBLAST is also equipped with a dot plot viewer, thus allowing researchers to create custom database for BLAST and run a dot plot similarity analysis within a single program. It is available to access to the NBLAST with http://nbitglobal.com/nblast.

## Introduction

Over the years, modern sequencing platforms have been generating genome information exponentially, and the number of genes with annotated function is increasing very rapidly as the bioinformatics field advances. Next-generation sequencing technologies have allowed researchers to obtain sequences from the whole genome or transcriptome easily in less time and in a cost-effective manner. Thus, effective bioinformatics tools are required to curate the obtained sequence data to match the annotated gene information in the public database. Since the introduction of the Basic Local Alignment Search Tool (BLAST) program in 1990 [1], this has been one of the most widely used bioinformatics programs. The algorithm of the BLAST program approximates sequence alignment by optimizing the local similarities between sequences by heuristic method. The BLAST algorithm has been further implemented into more advanced programs, such as WU-BLAST [2] and ScalaBLAST [3], but the most fundamental and earliest implementation of BLAST is by NCBI as a free web-based service, which is used by researchers worldwide. However, researchers cannot use their own sequence data with the web-based NCBI-BLAST program if the data exceed a size limit during the query sequence upload process, effectively precluding the analysis of large sequence data, such as whole transcriptome data sets. If researchers want to run BLAST on their own sequences, they will have to create a BLAST database in advance. However, NCBI-BLAST does not support custom database creation; hence, researchers must download the NCBI-BLAST program to analyze their own sequences. This introduces another barrier to accessibility, as executing the downloaded program requires a fairly advanced computational skill entailing the use of the command line interface.

The output of the BLAST program can be visualized using dot plots. A dot plot is a graphical representation of sequence similarities between two biological samples of proteins or nucleotides [4]. Dot plot is very efficient for analyzing of structural variations, such as duplication, inversion, and deletion between two sequences, as the results are displayed in a dot plot matrix. Dozens of dot plot programs are available online, but not many are integrated into a BLAST program in a standalone environment.

**Fig. 1.**
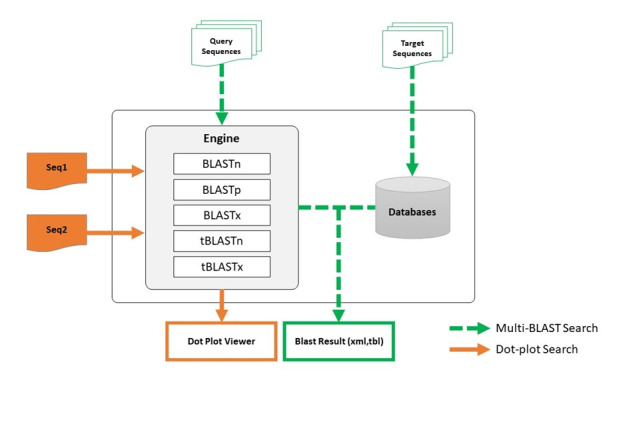
Flowchart of the NBLAST workflow incorporating BLAST analysis and a dot plot viewer.

NBLAST program is a graphical user interface (GUI)‒based BLAST program and dot plot viewer for similarity analysis between two sequences. Researchers can create their own database for a BLAST analysis and dot plot visualization. Because databases can hold a massive amount of data, researchers can analyze their own whole genome or transcriptome sequences using the NBLAST program with ease. Moreover, this program employs a two-way system that allows the created database to be used as either the ‘query’ or the ‘target’ sequence for BLAST analysis, which is not possible on the web-based NCBI-BLAST program. Prior to the current NBLAST program, Du et al. [5] reported a similar program, BlastGUI, that runs BLAST analysis using the two-way method. We improved the two-way BLAST analysis system by adding a dot plot viewing function in NBLAST, to enable researchers to carry out BLAST analysis on multiple sequences and generate a dot plot between two sequences in a single program. The flowchart for NBLAST workflow is shown in Fig. 1.

## Implementation

**Fig. 2.**
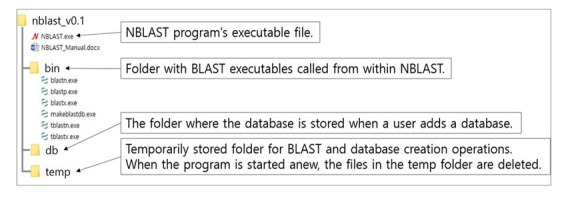
Program structure.

NBLAST is a GUI-based BLAST program. The user must input and directly execute the command on the nucleotide and protein sequence data. It is easy to run a BLAST analysis with simple mouse clicks. It also provides a built-in dot plot viewer to visualize the similarity analysis between two sequences. It also allows users to build custom databases to carry out BLAST analysis using their own sequences as queries. The program’s structure is shown in Fig. 2. NBLAST can only be used in the Windows environment. It is available to access to the NBLAST with http://nbitglobal.com/nblast.

### Creating database

**Fig. 3.**
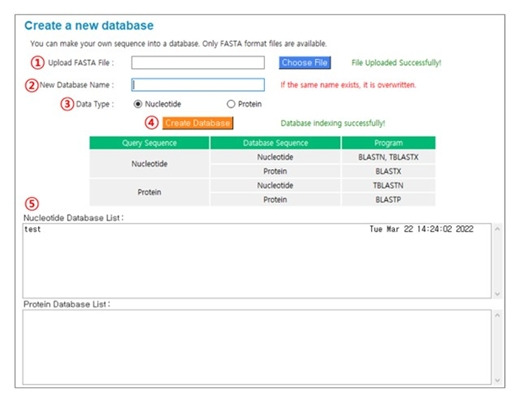
The procedure for creating a new database.

This is a function that allows users to create a custom BLAST database using their own FASTA format sequences. Fig. 3 shows the procedure to create a new database. (1) Upload ‘FASTA’ file: Click the “Choose File” button and select the sequence(s) you want to use to create a database. (2) New database name: The database name must be provided, and the file name of the selected sequence(s) is assigned by default. (3) Data type: Select “Nucleotide” or “Protein”. (4) Create database: Click the “Create Database” button to perform the indexing process to create a database. (5) Nucleotide Database List or Protein Database List: If the indexing process is successful, the newly generated database will appear in the list of previously registered databases.

### Removing databases

Deleting specific database(s) is not provided directly as a feature. To delete any specific database(s), access the “db” folder in Windows Explorer/File Explorer and delete all the files related to the named database directly. For example, if the database to be removed is a nucleotide sequence named “test’, the user must delete all four files (test, test.nhr, test.nin, test.nsq) from the “db” folder. Following this step, the deleted database will no longer appear in the database list.

### BLAST analysis

**Fig. 4.**
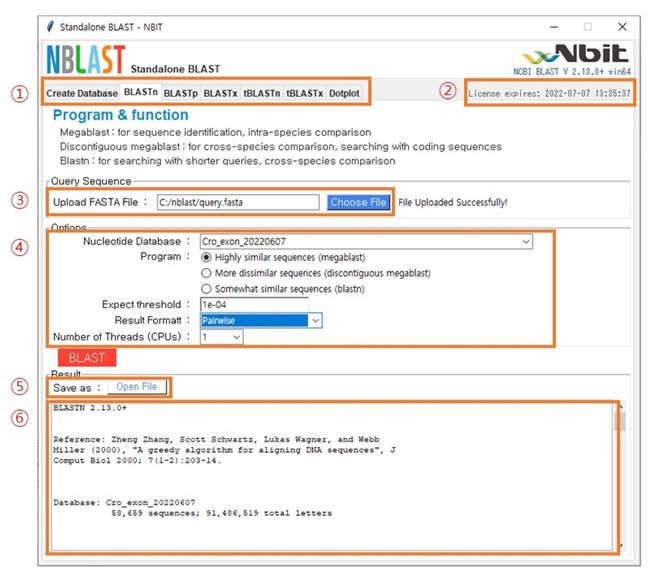
The structure of the main BLAST analysis window.

The main window of BLAST analysis is shown in Fig. 4. The main structure is as follows: (1) Program menu: You can choose five types of BLAST programs alongside the database creation menu. (2) The expiration date: This displays the expiration date of the user’s license. (3) Upload FASTA File: Click the “Choose File” button to select the sequence you want to analyze. (4) Options: The items are database selection, detailed option selection, and format selection for the result files. After every option is defined, click the “BLAST” button. (5) Open File: This “Open File” button appears when the analysis is run normally. Click the button to view on Notepad or WordPad. (6) Displaying the results: The bottom window shows the result. In order to obtain accurate results, it is necessary to proceed after confirming whether the query and database are nucleotides or protein sequences.

### Dot plot

**Fig. 5.**
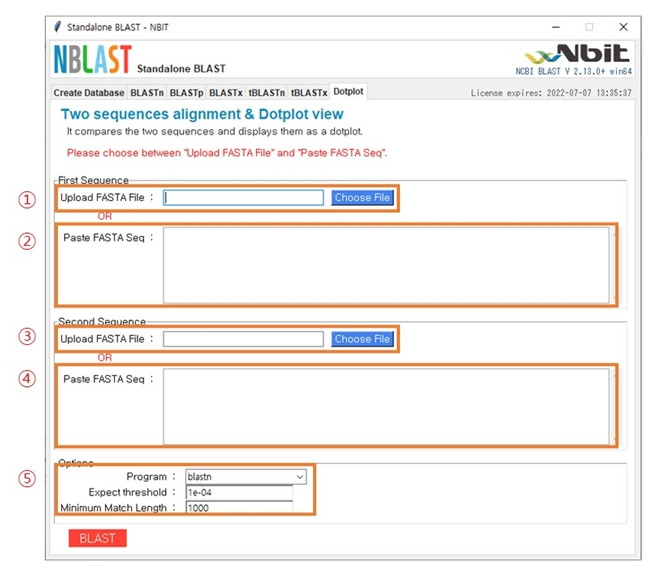
The structure of the main dot plot analysis window.

Fig. 5 shows the main window after selecting ‘Dotplot’ in the menu bar. There are two methods for data uploading. If you want to upload the data file directly, upload the FASTA file in the first sequence section in (1) and in the second sequence section in (3). Alternatively, if you want to upload the nucleotides or protein sequences, you can paste the sequences in (2) and (4). The options for the analysis can be set in (5). Depending on the type of sequences (nucleotides or protein), the filtering option of E-value and minimum match length can be set accordingly.

### Dot plot view

**Fig. 6.**
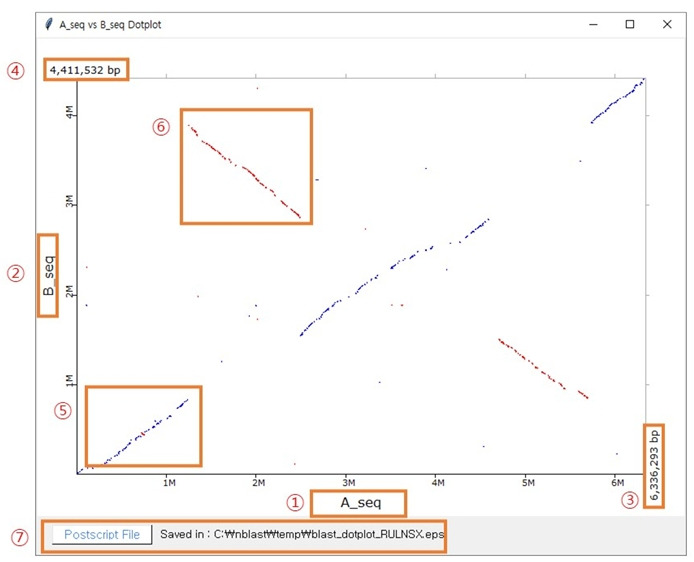
An example of a dot plot view.

An example of a similarity match between two sequences is shown in Fig. 6. The x-axis (1) and y-axis (2) are the sequences of A and B subjected to BLAST similarity matching respectively. (3) and (4) are the length of the sequences of A and B respectively. If the two sequences match in the forward orientation, the matching is displayed as blue dots (5). If the two sequences match in the reverse orientation, the matching is displayed as red dots (6). Results can be retrieved in the Postscript file in (7).

## Conclusion

NBLAST is a GUI-based BLAST program equipped with a dot plot viewer. Researchers often experience difficulty in the BLAST analysis of large data when using web-based BLAST programs and may want to create BLAST databases from transcriptomes or whole genome sequences. NBLAST is a two-system program that allows researchers to create custom databases for either ‘query’ or ‘target’ sequences to be used in the BLAST search. NBLAST also includes a function of dot plot visualization in addition to the BLAST search. Thus, researchers can carry out the BLAST analysis on multiple sequences as well as visualize similarity matches between two sequences as a dot plot in a single program.
